# Active regulatory elements recruit cohesin to establish cell specific chromatin domains

**DOI:** 10.1038/s41598-025-96248-4

**Published:** 2025-04-06

**Authors:** Emily Georgiades, Caroline Harrold, Nigel Roberts, Mira Kassouf, Simone G. Riva, Edward Sanders, Damien Downes, Helena S. Francis, Joseph Blayney, A. Marieke Oudelaar, Thomas A. Milne, Douglas Higgs, Jim R. Hughes

**Affiliations:** 1 MRC Molecular Haematology Unit, Weatherall Institute of Molecular Medicine, Radcliffe Department of Medicine, University of Oxford, Oxford, UK; 2 MRC WIMM Centre for Computational Biology, Weatherall Institute of Molecular Medicine, Radcliffe Department of Medicine, University of Oxford, Oxford, UK; 3 Max Planck Institute for Multidisciplinary Sciences, Am Fassberg 11, 37077 Göttingen, Germany; 4 Chinese Academy of Medical Sciences Oxford Institute, University of Oxford, Oxford, UK

**Keywords:** Molecular biology, Epigenetics, Nuclear organization

## Abstract

As the 3D structure of the genome is analysed at ever increasing resolution it is clear that there is considerable variation in the 3D chromatin architecture across different cell types. It has been proposed that this may, in part, be due to increased recruitment of cohesin to activated cis-elements (enhancers and promoters) leading to cell-type specific loop extrusion underlying the formation of new sub-TADs. Here we show that cohesin correlates well with the presence of active enhancers and that this varies in an allele-specific manner with the presence or absence of polymorphic enhancers which vary from one individual to another. Using the alpha globin cluster as a model, we show that when all enhancers are removed, peaks of cohesin disappear from these regions and the erythroid specific sub-TAD is no longer formed. Re-insertion of the major alpha globin enhancer (R2) is associated with re-establishment of recruitment and increased interactions. In complementary experiments insertion of the R2 enhancer element into a “neutral” region of the genome recruits cohesin, induces transcription and creates a new large (75 kb) erythroid-specific domain. Together these findings support the proposal that active enhancers recruit cohesin, stimulate loop extrusion and promote the formation of cell specific sub-TADs.

**Supplementary Information:**

The online version contains supplementary material available at 10.1038/s41598-025-96248-4.

## Introduction

During interphase, the mammalian genome is organised into a series of hierarchical structures ranging from chromosome territories to smaller domains including active (A) compartments, inactive (B) compartments, lamina associated domains (LADs) and topologically associating domains (TADs). While each of these was originally described at relatively low resolution and in a limited number of cell types, as many more cell types have been examined at increased resolution, finer levels of structure became apparent. These structures are dynamic and vary in a cell-type specific manner. Although the structure of TADs was originally considered to be relatively constant across cell types, at higher resolution these TADs and sub-TADs can be seen to vary during differentiation, development and in the cell cycle^1–5^.

It is now generally accepted that TADs are formed, in part, by the process of cohesin-mediated loop extrusion^6–9^. Once recruited, cohesin can translocate across chromatin until it reaches a CTCF boundary element with the N-terminus of CTCF orientated towards the translocating cohesin, at which point further extrusion is impeded and cohesin accumulates at this site before it is off loaded by WAPL^10–13^. Cohesin is a multi-subunit protein complex which does not have a specific DNA binding motif, instead current evidence suggests that cohesin is loaded onto chromatin facilitated by the NIPBL-MAU2 complex^14,15^, although there is debate about where cohesin is loaded. Extensive studies removing the key players (Cohesin, NIPBL, WAPL, CTCF) have suggested a model in which TADs result from recruitment of cohesin across the entire genome, delimited by the distribution of CTCF sites^16^. Much less is known about how the cell-type specific sub-TADs form and the role that they play in mediating enhancer-promoter interactions. It has previously been noted that cohesin is enriched at enhancers within activated sub-TADs^17–20^ and there is evidence suggesting a link between the recruitment of cohesin via its interaction with NIPBL and gene regulatory activity^17,21–27^, which would go some way to providing a mechanism for cell-type specific domain formation.

Here we investigated if active enhancers are sites of cohesin recruitment to chromatin and asked if this underpins the formation of sub-TADs. First, we took advantage of natural variation in human enhancer sequences that alter activity. Using a genome-wide approach we identified enhancers whose activity is completely lost or gained due to sequence polymorphisms and showed that cohesin recruitment is coherently lost or gained. To increase the number of observations beyond this small number of absolute losses or gains, we used allelic skew analysis of the more frequent heterozygous regulatory variants to judge the relationship between the degree of regulatory activity and cohesin recruitment. This approach allows for a precise comparison between the levels of cohesin recruitment and the corresponding degree of regulatory activity of each allele. Assays were evaluated in the same cell type when one allele is altered by a regulatory variant. This showed a strong positive correlation between RAD21 occupancy and regulatory activity, particularly when considering elements defined as enhancers based on their chromatin profiles.

To examine this experimentally, we studied the alpha globin cluster which is contained within a 165 kb TAD found in all cell types tested, whereas a 65 kb evolutionarily conserved, erythroid-specific sub-TAD, containing all alpha-like genes and five erythroid-specific enhancers (R) in the order 5’ R1-R2-R3-Rm-R4-ζ-α1-θ1-α2-θ2 3’ forms during erythroid differentiation. This sub-TAD starts to form early in erythroid differentiation when the alpha globin is starting to be transcribed at low levels, at which time the cis-regulatory elements are open and bound by erythroid specific transcription factors (TFs)^28,29^. When alpha globin transcription is fully established in mid-late differentiation, the enhancers and promoters are in close proximity. Of interest, we have previously shown that there is enrichment of both cohesin and NIPBL at the alpha globin enhancers during erythroid differentiation^21,30^. Acute degradation of cohesin during the early stages of differentiation reduces alpha globin expression and inserting CTCF sites between the enhancers and promoters strongly decreases alpha globin expression when the N-terminus of these sites prevents translocation of cohesin from the enhancers to the promoters^31,32^. Together these findings suggest that recruitment of cohesin to the activated enhancer elements and its translocation from the enhancers to the promoters plays a part in formation of the subTAD^33,34^ .

In support of this hypothesis, here we show that removing all enhancer elements (R1, R2, R3, Rm, R4) from the alpha globin locus severely decreases cohesin recruitment, and decreases the formation of a cell type-specific sub-TAD. The sub-TAD was partially reformed by adding back the strongest enhancer element (R2) to its natural position in the alpha globin locus. This showed that some degree of enhancer activity is required to form the sub-TAD but did not show if this was an intrinsic property of the enhancer or if formation of the sub-TAD also required other features of the alpha globin locus.

To address this we repositioned the strong R2 enhancer away from its normal environment in an apparently featureless region of the genome to ask if a single enhancer alone is sufficient to recruit cohesin to an artificial locus and form a sub-TAD. To do this and remove all confounding elements and effects from the native environment, we identified an apparently “neutral” region of the genome on mouse ChrX and inserted the isolated R2 enhancer element into this region: this showed that R2 alone is sufficient to recruit cohesin and create an erythroid-specific chromosomal domain in this artificial locus. Together these experiments show that a single active enhancer, on its own, can recruit cohesin and form an erythroid-specific sub-TAD.

## Results

### Cohesin colocalises with active enhancers

**Fig. 1:**
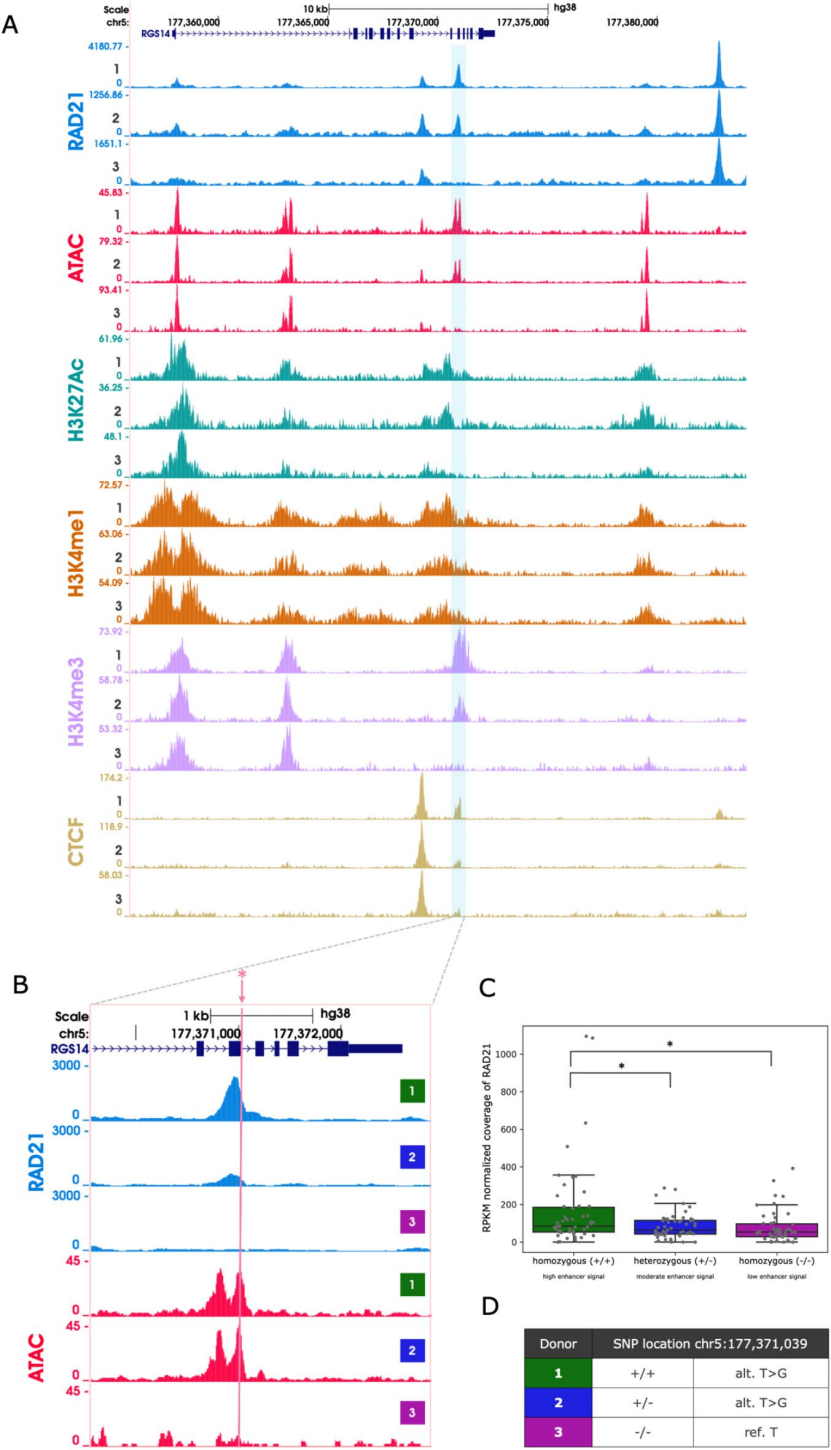
The presence of enhancers can vary naturally in a population. Sequences showing the characteristic chromatin marks of enhancers are positively correlated with cohesin occupancy. (**A**) An example of a site with natural variation of enhancer signal across the three donors and the correlation with cohesin occupancy. Region shown is chr5:177,367,955 − 177,373,964 (hg38). Numbers 1, 2, 3 on each track indicate the anonymized donor identifiers. ChIP-seq for RAD21, CTCF and histone marks (H3K4me1, H3K4me3, H3K27ac) are shown for each donor in the top panel along with the open chromatin signal (ATAC-seq). The region highlighted in blue is shown in detail in (B). (**B**) Zoom in on the enhancer peak where the signal across the donors varies according to the genotype: donor 1 is homozygous (+/+) and displays a positive ATAC-seq signal and the greatest peak of RAD21, donor 2 is heterozygous (+/−) and displays a positive ATAC-seq signal and a moderate peak of RAD21, donor 3 is homozygous (−/−)with no ATAC-seq or RAD21 signal. The pink line indicates the SNP we have identified as potential causal for this difference across the donors. The UCSC Genome Browser (http://genome.ucsc.edu) was used for visualization of ATAC-seq, ChIP-seq and gene annotations tracks in both (A) and (B). (**C**) There is a positive correlation between the presence of enhancer signal and coverage of RAD21 suggesting active enhancers accumulate cohesin. For 51 regions in which there is a robust difference in ATAC-seq signal across the donors, RAD21 signal was calculated for the three donors and the donors classified as either homozygous +/+ signal (green), homozygous −/− (purple), or heterozygous (blue). Each of the 51 individual data points are plotted as grey dots. Statistical significance between pairs was calculated using the paired T-test, asterisk indicates p-value ≤ 0.05. Coverage was normalised by RPKM and by region size. Plotted using matplotlib (v3.4.3) library in python (v3.10.7) (**D**) Table indicating the SNP location for this example, and the variant and genotype associated with each donor. Additional examples are shown in Fig. S1–S2.

It has been previously proposed that cohesin is loaded at active regulatory elements across the genome^17,21–27^. Although to our knowledge this has not been quantified on a genome-wide scale. To investigate this in detail, we selected three donors at random from the Oxford Biobank (which throughout will be referred to as donor 1, 2 and 3), and performed histone modification ChIP-seq for H3K27Ac, H3K4me1 and H3K4me3, along with CTCF ChIP-seq and ATAC-seq in CD34 + erythroid cells (Fig. 1A). This characterised the regulatory landscape in each individual and the open chromatin sites detected by ATAC-seq were classified based on their associated chromatin marks.

We first applied REgulamentary^35^ on the chromatin datasets from the 3 donors. REgulamentary is a rule based bioinformatics tool that uses the varying ChIP-seq enrichment of key chromatin modifications (H3K4me3, H3k4me1, H3k27ac and CTCF) around called open chromatin sites to classify them into separate cis-regulatory classes (promoter, enhancer and CTCF sites or promoter or enhancers bound by CTCF) (Fig. S1–S3). Using the phased genome data from these 3 individuals we detected specific haplotypes overlapping regulatory regions and used these to determine whether skewed regulatory activity existed between the two haplotypes in any given individual (Fig. S4 and S5). We then performed RAD21 ChIP-seq in CD34 + erythroid cells from these three donors to determine whether there was a correlation between enhancer activity and cohesin recruitment.

Using stringent cut-offs that identified extreme skew in enhancer activity (detailed in Materials and Methods), we identified 51 examples of polymorphic enhancers in which the associated enhancer was either active or inactive based on open chromatin and active chromatin marks. We subsequently performed the same skew analysis on these extreme regions using RAD21 ChIP-seq data from these individuals to determine whether cohesin showed similar skew. Figure 1A shows an example from this meta-analysis in which the loss of a regulatory element leads to the loss of cohesin recruitment at the same site. Figure 1B shows a zoom in on the affected cohesin peak and shows the coordinated loss of an open chromatin signal and cohesin recruitment from a fully active homozygous reference individual, the decreased signal in a heterozygous individual to the complete loss in a homozygous variant individual. Combining the analysis across these 51 regions of extreme skew showed a statistically robust difference in the amount of RAD21 present at these sites, linked to the presence of a damaging regulatory variant both in heterozygosity or homozygosity (Fig. 1C and D).

We then extended this approach using allele-specific analysis to evaluate skew of markers of activity genome-wide across all promoter, enhancer and CTCF sites and correlated this with skew in RAD21 signal (Fig. S6). This shows a positive correlation between the respective activity or binding signals in each class. For the CTCF class of elements a positive correlation would be expected as the degree of CTCF binding would correlate with the degree of loop extrusion blocking, and the amount of RAD21 signal that accumulates at any given CTCF site (Fig. S6, CTCF skew vs. Rad21 skew in CTCF blue column). In agreement with this analysis of the motifs around variants associated with skew in CTCF sites show a strong enrichment for variants of the canonical CTCF motif, which is decreased in the presence of a down skewing variant (Supplementary Table S6). In contrast, motif analysis of skew associated variants in promoter elements showed an enrichment for a wide range of GC and CG rich motifs, such at the KLF, ETS and SP families, presumably driven by the typical GC content of mammalian promoters. The frequency of these motif are also decreased in the presence of down skewing variants (Supplementary Table S6). Variants in enhancer elements show an enrichment for a wide range of TF binding motif, in agreement for the generally enriched TF binding of these elements and included important binding site for erythroid TFs such as GATA, Tal1 and Jun and Fos motifs (which are bound by NFE2 in erythroid cells). Similar to both CTCF elements and promoters the frequency of these motifs are decrease in the presence of down skewing variants (Supplementary Table S6). Importantly, a similar striking positive correlation is also seen between the appropriate active marks for promoters (H3K4me3, ATAC and H3K27ac) and enhancers (H3K4me1, ATAC and H3K27ac) with RAD21 signal, with a greater correlation at enhancer elements (shown in green, Fig. S6) than promoter elements (shown in yellow, Fig. S6). Therefore, by leveraging natural human variation we can show that the degree of regulatory activity at both enhancers and promoters, but especially at enhancers, is linked to the level of recruitment of cohesin to these sites and that this can be substantially altered by naturally occurring regulatory variants.

Although highly supportive of the hypothesis that regulatory activity can recruit cohesin for loop extrusion, this analysis only shows correlation between these two genomic processes but does not prove causation. A further limitation of this analysis is that the elements that can be defined as enhancers using chromatin marks are typically only putative enhancers. Therefore, to investigate this hypothesis more rigorously, we turned to a highly characterised locus in which genetically defined enhancer-like elements could be studied in defined cell types that allow it to be observed in both an active and an inactive state.

### The alpha globin super-enhancer as a tractable model to controllably test cohesin recruitment to active enhancers

**Fig. 2:**
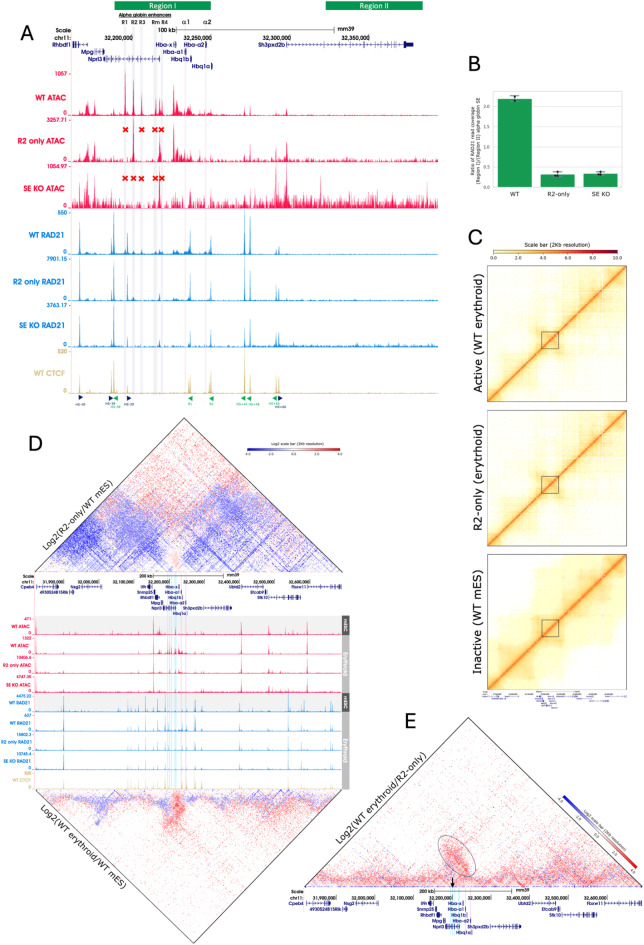
Cohesin accumulates at the sites of active enhancers from which chromatin contacts arise. (**A**) ATAC-seq and RAD21 ChIP-seq spanning the alpha globin locus (chr11:32,146,649 − 32,280,800, mm39). Alpha globin enhancers are highlighted in blue and the two alpha genes (α1 and α2) highlighted in pink. GENCODE VM32 genes within the region are shown at the top in dark blue. ATAC-seq and ChIP-seq tracks for RAD21 and CTCF are shown with corresponding model indicated to the left. Red crosses above tracks indicate which of the enhancers are knocked out. All bigwig tracks are RPKM normalised and plotted with bin size 1. The orientation of CTCF motifs are shown below the CTCF track in purple (forward) and used plotting the coverage in (B). (**B**) Cohesin residency within the region of the alpha globin SE is significantly reduced upon deletion of the enhancer elements. Bar plot showing the ratio of cohesin coverage inside (Region I in (A), chr11:32,188,452 − 32,249,902) versus outside (Region II in (A), chr11:32,300,054 − 32,391,239) the alpha globin SE region. Two replicates for WT and three replicates for the R2-only and SE KO model are plotted; each point represents one replicate. Error bars represent standard deviation across replicates. Plotted using matplotlib (v3.4.3) library in python (v3.10.7). (**C**) Tiled-C plots for the region chr11:31,818,001–32,690,000 (mm39), where the alpha-globin enhancers are active (erythroid cells), only the R2 enhancer is present and active (erythroid cells), and inactive (undifferentiated mES cells). Contact maps are present at 2 kb resolution. GENCODE VM35 track is shown below the three subplots. Marked by black squares is the region chr11:32,154,606 − 32,260,344 which containing the active region within the sub-TAD. (**D**) Activation of erythroid specific alpha globin enhancers coincides with a change in 3D domain structure. Figure shows comparison of the contact frequencies in the different models presented in (C). Upper triangle: interaction matrix for log_2_(R2/WT mES). Lower triangle: interaction matrix for log_2_(WT erythroid/WT mES), here the inactive alpha globin locus in ES cells is compared to the model in which only the R2 enhancer is present and active. Tiled-C in WT mESC was generated for inactive enhancers, in WT fetal liver for active enhancers and R2 only fetal liver model for R2 only. In both interaction plots data from the two states were normalized before plotting a log2 comparison of the chromatin interactions. ATAC and RAD21 ChIP-seq tracks are from the corresponding models, CTCF ChIP-seq is from WT mESC for the inactive model and WT embryoid body erythroid cells for the R2 only model. (**E**) The active alpha globin locus in WT Ter119 + fetal liver cells is next compared to the model in which only the R2 enhancer is present and active. Data from the two states were normalized before plotting a log2 comparison of the chromatin interactions across the locus in a fully active versus R2 only active states. Black arrow indicates the R2 enhancer, black oval highlights the region in which interaction frequencies are enriched in the WT over the R2 only model. Note: Datasets shown in C and D have been normalized in a pairwise manner for log_2_(R2/WT mES), log_2_(WT erythroid/WT mES) and log_2_(WT erythroid/R2) to account for the differences in sequencing depth across experiments. The UCSC Genome Browser (http://genome.ucsc.edu) was used for visualization of ATAC-seq, ChIP-seq and gene annotations tracks in (A), (C), (D) and (E). Tiled-C plots were created using HiCPlotter^43^ (v0.6.6).

The clusters of both human and mouse alpha globin enhancers (R1-R4) fulfil the definition of super-enhancers^34,36,37^. This locus is not only one of the best studied regulatory regions of the genome, there also exists several robust cellular models in which to study the locus in both its active (erythroid) and inactive (non-erythroid) states. To study the recruitment of cohesin to these enhancer elements, we made use of two previously described large-scale mouse models engineered using synthetic biology: the first in which all of the five enhancers have been deleted (super-enhancer knock out (SE KO)^33^) and another model in which all enhancer elements except for the strongest of the five component enhancers of the super-enhancer (R2) have been deleted (R2-only^33^). To study the enhancer activity in these models, CD71 + erythroid cells from embryoid body cultures^38^ and Ter119 + primary erythroid cells from mice were obtained to study the SE KO and R2-only models respectively (refer to Table S4 for full list of models used in this study). RAD21 ChIP-seq was then performed on the SE KO, R2-only and WT control CD71 + erythroid cells. Peaks of cohesin were found in the WT erythroid cells at all five enhancer elements (Fig. 2A, see positions of elements R1-4 and Rm highlighted by blue bars in RAD21 ChIP-seq tracks (blue)). By contrast, in the SE KO model, no peaks of cohesin were found at any of the enhancer elements. Importantly, in the R2-only model cohesin was only observed at the site of the remaining R2 enhancer element. In both models, similar peaks of cohesin at the CTCF sites could be seen as those observed in WT cells. By visual inspection, it appeared that between the two convergent CTCF sites containing the alpha globin sub-TAD (HS-39 and θ2) there is a greater build-up of cohesin across the domain in the WT compared to both enhancer KO models (Fig. 2A). To quantify this observation, the total coverage of RAD21 ChIP-seq signal across the region between the convergently orientated CTCF sites flanking the sub-TAD (labelled ‘Region I’ in Fig. 2A) was compared with a nearby size-matched region (~ 61.5 kb) outside of the CTCF sites (labelled ‘Region II’ in Fig. 2A) and the ratio of the signal calculated. Figure 2B shows that cohesin across the SE region is enriched ~ 6 fold in the WT compared to the enhancer KO models. This shows that in the presence of all 5 alpha globin enhancers, recruitment of cohesin across the entire sub-TAD is substantially increased relative to when these enhancers are knocked out.

### Active enhancers give rise to tissue-specific sub-TADs

Cohesin is known to play an important role in the establishment of 3D genome structure through its role in loop extrusion^39–42^. Since fully or partially deleting the SE changes the amount of cohesin recruited to the active alpha globin sub-TAD in erythroid cells we addressed how this might impact the 3D genome structure of this locus. We performed Tiled-C chromosome conformation capture in undifferentiated mESCs in which the alpha globin locus is inactive and genes are not transcribed, and compared this to a fully activated state in WT erythroid cells derived from mouse fetal liver (Fig. 2C). When plotted as a log2 comparison plot, increase in interaction frequencies across the alpha globin locus can be readily observed in the active locus (Fig. 2D lower heatmap). Next, we looked at how chromatin interactions would change when four of the five enhancers are deleted to leave only the R2 element, comparing these patterns in erythroid cells^33^ and inactive mESCs (Fig. 2C and D upper heatmap). This analysis showed that while a greater degree of interaction is detected within the sub-TAD when all enhancers are present compared to only the single R2 enhancer (region marked by black oval in Fig. 2E) there is still a pronounced increase in the interaction frequencies around the alpha globin locus in the R2-only erythroid model compared to the inactive mESCs.

These data demonstrate that even a single active enhancer can direct the recruitment of cohesin to the alpha globin cluster and influence its 3D structure via loop extrusion albeit with a reduced capacity compared to when all native enhancers are active. We conclude that the number of active enhancers controls the recruitment of cohesin within the domain and directly affects the degree of 3D interaction.

### Identification of a neutral but activatable region of the genome

An important limitation of using the enhancer KO models to investigate the independent role of an enhancer in recruiting cohesin to the cluster is that it is impossible to control for all potentially confounding factors that may contribute to the recruitment of cohesin and 3D genome organisation at this locus. Whilst the R2-only and SE-KO models provide useful insights into how an enhancer might influence cohesin recruitment, the results may be influenced by activity from neighbouring active genes, and/or other regulatory elements within or surrounding the locus. Additionally, previous analysis of sequence conservation across this locus have identified multiple regions of sequence conservation, the function of which still remain unknown^44^. We therefore developed an orthogonal approach to study cohesin recruitment by the R2 enhancer which avoids any potentially confounding influence from such elements by inserting this element outside of its normal chromatin context, in a region of the genome which is apparently devoid of gene or regulatory activity. We therefore first needed to identify a suitable region of the genome in which to insert the R2 enhancer to determine if this element is capable of recruiting cohesin in an independent manner. The target locus needed to be amenable to activation: therefore, the search was limited to regions within the A compartment of the genome^45^ to avoid inserting into repressive LADs. To classify the A and B regions, compartmentalisation analysis was performed on published Hi-C data in mESCs^46^. We chose to limit our search to chrX, to increase the efficiency of the genome editing strategy by making use of a male mESC line which circumvents the need to isolate homozygously edited clones. Once we had identified an activatable region, we analysed ChIP-seq and ATAC-seq data from Ter119 + primary erythroid cells and mESCs to assess the chromatin landscape. To avoid confounding effects, whilst the region needs to be activatable (i.e., not epigenetically repressed), the ideal region should be as ‘neutral’ as possible. We therefore looked for a region devoid of transcriptionally repressive marks (H3K9me3 and H3K27me3), open chromatin signal (determined by ATAC-seq), ChIP-seq signals for histone modifications typically associated with active enhancers and promoters (H3K4me1 and H3K4me3 respectively) or annotated genes. Of the candidate regions on chromosome X, one satisfied all criteria for being potentially neutral and activatable chromatin in both mESCs and primary erythroid cells: an approximately 110 kb region at chrX:11,224,970 − 11,335,361 (mm39) (Fig. S7). To ensure there was no underlying complex chromosome structure other than the normal proximity signal expected of the chromatin fibre in this region, DpnII fragments ranging between 1 and 3 kb in length were identified across the locus to act as viewpoints of interest, and capture oligonucleotides were designed to six viewpoints across the locus (Table S1). Capture-C was then performed across this locus in wild type mESCs and CD71 + erythroid cells isolated from embryoid body cultures, this generated interaction data of a higher resolution than the available Hi-C data and confirmed the absence of any recognisable structure in the two cell types (Fig. S8). One of these DpnII viewpoint fragments was subsequently used as a ‘landing pad’ (LP) in which to target the R2 alpha globin enhancer element, giving the ability to assay local chromatin structure pre- and post-editing with the same set of capture oligonucleotides to allow for direct comparison of interaction profiles.

### Inserting the R2 enhancer into a neutral region of the genome results in the formation of a new erythroid-specific domain structure

**Fig. 3:**
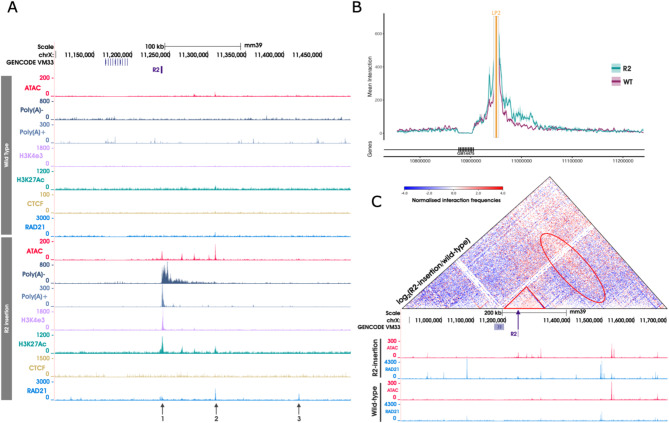
Insertion of a 325 bp sequence containing the R2 alpha globin enhancer into a neutral region of the genome, initiates significant changes when activated by erythroid differentiation. (**A**) In erythroid cells, the insertion of the R2 enhancer element gives rise to novel peaks of open chromatin, H3K27Ac, RAD21 and *de novo* transcription. All data were generated in erythroid material derived from embryoid body culture. Top panel shows the data generated in wild-type cells, bottom panel shows data from R2-insertion model cells. Arrows highlight the RAD21 peaks discussed in the text. A putative CTCF motif identified to overlap RAD21 peak 3 by FIMO analysis, however no signal seen by CTCF ChIP-seq. (**B**) The inserted R2 element contacts sites upstream and downstream suggestive of domain formation. Comparison of the local genome topology around the R2 insertion site in R2-chrX erythroid cells versus WT. NG Capture-C interaction profile from the R2 (orange line) with a 1 kb exclusion zone around the viewpoint. WT profile is shown in purple and R2-chrX in turquoise. Each profile represents normalised, averaged unique interactions from three biological replicates. Standard deviation is represented as a halo around the average across a 3 kb sliding window. Genomic location and relative positioning of genes is shown below the interaction profile. Capture-C plot was created using CaptureCompare (see Methods section for details). (**C**) The R2 enhancer can initiate the formation of a sub-TAD when isolated outside of its usual chromosome context. Tiled-C heatmap showing the normalized interaction frequencies at 2 kb resolution across region chrX:10,875,000–11,705,000 (mm39). Arrow indicates the site in which R2 is inserted. Data is presented as a log_2_ comparison plot of two normalized Tiled-C experiments. Triangle contains a region of increased interactions when the R2 insertion model is compared to the WT and indicates the formation of a new sub-TAD. The oval outline indicates an additional stripe of newly formed interactions which suggest the activated elements within the domain are also forming longer-range interactions beyond the newly formed sub-TAD. Tandem repeats of the H2al1 genes result in multi mapping issues which explains the lack of signal detection from the region surrounding these genes. The UCSC Genome Browser (http://genome.ucsc.edu) was used for visualization of ATAC-seq, RNA-seq, ChIP-seq and gene annotations tracks in (A) and (C). Tiled-C plots were created using HiCPlotter^43^ (v0.6.6).

A 325 bp segment of DNA containing the sequence for the R2 alpha globin enhancer was targeted to the second landing pad (LP2) in the chrX locus in mESCs using CRISPR-Cas9-mediated homology directed repair (HDR). This edited cell line is referred to as ‘R2-insertion’ model. To determine whether the inserted R2 became accessible and caused changes in local chromatin in ESCs, ATAC-seq was performed on R2-insertion and WT ESCs. R2 is a well characterised erythroid-specific enhancer, therefore in undifferentiated ESCs, in which the enhancer is inactive, no change in accessibility was observed as expected (Fig. 3A). R2-insertion and WT mESCs were then differentiated via organoid culture to form embryoid bodies and provide a source of CD71 + erythroid cells which were isolated after seven days of differentiation, at which point the R2 enhancer is expected to be active, and ATAC-seq performed.

ATAC-seq reads from this cell line were aligned to a custom reference genome allowing for multi-mapping of reads that mapped to both the native R2 and the inserted R2 sequence. Reads that originated directly from the 325 bp R2 sequence were randomly mapped to one of the two genomic locations harbouring R2 resulting in peaks at both locations. To confirm that the accessible chromatin peak over the inserted R2 was a true signal, rather than a false-positive signal originating from native R2, the profiles of the paired-end reads spanning the junctions of the R2 insertion, and so unique to the edited locus, were compared to the unique junctions at the native R2 (Fig. S9A). This junction analysis, combined with an overall increase in the mapping of the internal R2 reads that did not span a junction (Fig. S9B) confirmed that the R2 element edited into the Chr X locus was indeed open chromatin in erythroid cells. Importantly, the activation of the R2-insertion cells by erythroid differentiation, resulted not only in local changes in accessibility in the vicinity of the insertion on chromosome X, but also caused the activation of distal regions causing the formation of novel ATAC-seq peaks up to ~ 75 kb downstream of the insertion site even though the sequence of these sites were the same in both edited and unedited cell (Fig. 3A). These results show that the inserted R2 enhancer element exerts changes on surrounding chromatin over a substantial distance suggesting the establishment of long-range interactions upon its insertion. Together these findings show that the ectopically inserted R2 sequence acts as a long-range cis-regulatory element.

H3K27ac is associated with active chromatin and is found at both enhancers and promoters, H3K4me3 is predominantly associated with active promoters and H3K4me1 is predominantly associated with active, or primed enhancers. ChIP-seq for the histone marks H3K27ac, H3K4me1, and H3K4me3, were performed in the R2-insertion model and WT erythroid cells to characterise any changes in the regulatory landscape following the insertion of R2 (Fig. S10 and S11). The novel ATAC-seq peaks appearing downstream of the R2-insertion in erythroid cells were marked with H3K27ac (Fig. 3A) and H3K4me1 (Fig. S11). Interestingly, unlike the downstream elements, the inserted R2 sequence itself was also marked with H3K4me3 in erythroid cells (Fig. S11). This was unexpected given the histone marks observed at R2 in the native alpha globin environment are typical of an enhancer element and enriched for H3K4me1 (Fig. S10B). Therefore, unexpectedly, the inserted R2 enhancer had the chromatin signature of an active promoter whereas the newly formed accessible regions of chromatin lying downstream of the inserted R2 had the signatures of enhancers (Fig. S12).

A further important question was: what was the nature of the distal sequences that were activated by the inserted R2 element? The initial criteria in the search for targetable regions in the genome was based on the detection of open chromatin and CTCF sites, which where all absent from our final artificial locus. However, careful retrospective analysis of the regions that are activated by R2 in the artificial locus showed them to be weakly marked by H3k4me1 even in unedited erythroid cells and that this signal increases when R2 is inserted upstream and that they also acquire H3k27ac marks (Fig. S11). This suggests that there is some low-level cryptic activation at these sites supported by the existence of erythroid specific TF motifs such as GATA1 which are found within these regions. This cryptic activity is then increased to form detectable open chromatin sites under the influence of the inserted active R2 element.

Given that the newly inserted R2 enhancer element in R2-insertion erythroid cells had the signature of an active promoter, we asked whether this initiated RNA transcription. Strand specific Poly(A)- and Poly(A) + RNA-seq was performed on R2-insertion and WT erythroid cells (Fig. 3A). In both Poly(A)- and Poly(A) + data, unidirectional transcription was observed. In Poly(A)-, transcription was seen from the R2 insertion site, producing a ~ 75 kb nascent transcript including the regions containing the novel enhancer-like elements; and in Poly(A)+, a ~ 4 kb stable transcript from the inserted R2 was observed. Together, these data show that the inserted R2 in the R2-insertion model acts as a promoter-like regulatory element producing both non-polyadenylated and polyadenylated, unidirectional transcripts and activating cryptic elements over a region of 75 kb.

### The alpha globin R2 enhancer sequence is sufficient to form a regulatory domain in erythroid cells

To investigate whether the insertion of R2 altered the 3D chromosome conformation in and around the insertion site, Capture-C was performed from the viewpoint of LP2 (R2 insertion site) in both R2-insertion and WT erythroid cells. As expected, in WT erythroid cells Capture-C showed a normal distribution of interactions around the viewpoint, consistent with the proximity signal of a chromatin fibre with no underlying regulatory structure (Fig. 3B). By contrast, in the R2-insertion erythroid cells showed increased interactions predominantly between the inserted R2, and the region downstream (3’) extending for 75 kb including the novel enhancer-like elements. Capture-C interactions were not specifically focused at the novel enhancer-like elements, but rather extended over the entire domain encompassing the region of active transcription. These findings suggest that insertion of the R2 element might promote the formation of a new cell-specific chromatin domain. However, Capture-C only assesses interactions from one viewpoint (LP2) and therefore could not assess the formation of higher order structures such as TADs and subTADs. To investigate the 3D interactions across the whole of the locus in an unbiased manner, Tiled-C^1^ was performed across a ~ 3.3 Mb region spanning the inserted R2 in the R2-insertion and in WT erythroid cells (Fig. 3C). In agreement with the Capture-C data, Tiled-C data revealed an increase in the interaction frequencies of the R2 insertion site with the surrounding locus in the edited cells compared to WT, reminiscent of a small TAD or sub-TAD-like structure.

### Activation of the R2 erythroid enhancer is linked with cohesin recruitment

We hypothesised that proteins may be loading at, and translocating from, the newly inserted R2 element and subsequently directing loop extrusion and long-range chromatin interactions. Although transcription per se has been implicated in this process, there is now abundant evidence that the cohesin complex plays the major role in loop extrusion. To analyse the binding of cohesin at this engineered locus, we performed ChIP-seq of the cohesin component RAD21. The endogenous RAD21 protein was tagged with a twin Strep-tag (RAD21^TST^) and ChIP-seq was performed in R2-insertion and WT erythroid cells Fig. 3A (also Fig. 3C). A peak of cohesin is observed at the R2 insertion site in R2-insertion erythroid cells (arrow 1 in Fig. 3A), and this corresponds to the open chromatin site identified through ATAC-seq. This is consistent with the newly inserted R2 element acting as a recruitment site for cohesin as reported for other enhancers and promoters^17,21–27^.

The process of cohesin-mediated loop extrusion is usually delimited by CTCF binding sites and enriched peaks of cohesin appear at these sites when the translocation of cohesin is stalled by CTCF. Other mechanisms which stall the translocation of cohesin have been reported^27,47,48^. While CTCF ChIP-seq data in both WT erythroid cells and WT mESC did not show any significant peaks in this domain (Fig. 3A) we observed a strong cohesin peak 71 kb downstream of the enhancer (arrow 2 in Fig. 3A). This coincided with the previously identified ATAC peak that appears at the R2 insertion site and chromatin marks suggest it to be an active enhancer like element at this site rather than a CTCF element. However, using FIMO analysis, a putative negatively oriented CTCF binding site was found to overlap the cohesin peak at the most downstream open chromatin site ~ 182 kb downstream of the inserted R2 sequence (chrX:11,420,541 − 11,420,558, arrow 3 in Fig. 3A). To confirm that the activity of the inserted R2 element did not cause binding of CTCF at this cryptic binding site in the edited erythroid cells CTCF ChIP-seq was performed however no significant peaks of CTCF were identified in this region. Similarly, no CTCF peak was seen at a second RAD21 peak downstream of R2 (arrow 2 in Fig. 3A). Such peaks of cohesin not associated with CTCF sites have been previously noted^49^: it has been suggested that a small fraction of cohesin does not colocalise with CTCF and instead resides at sites marked by active chromatin features and bound by cell type specific TFs^17,18,50^, which could be the case in the R2-insertion model. However, these results clearly show that the insertion of a single well characterised enhancer into our defined neutral region is sufficient on its own to cause a substantial increase in cohesin recruitment to chromatin at sites across a 182 kb domain of previously featureless genome.

## Discussion

In this work we have presented three different approaches to studying the recruitment of cohesin to active enhancer elements. It has been recently shown that single nucleotide mutations can give rise to *de novo* enhancers in brain^51^, with other examples describing how sequence variations can disrupt transcription factor motifs and significantly impact the enhancer landscape^52^. We speculated that common variants in the form of SNPs that vary between individuals may be correlated with the presence or absence of enhancer activity and could therefore be used as naturally arising examples in which to study how cohesin recruitment varies with enhancer activity. Characterising three normal individuals we have used rare but extreme examples of regulatory variation in combination with much more frequent but more subtle variants to demonstrate the positive correlation between enhancer activity and cohesin occupancy genome wide. While a small fraction of these enhancer elements also bind CTCF, the majority do not, therefore these data suggest that the enhancer activity itself somehow influences the recruitment or engagement of cohesin with chromatin. We therefore used a model in which we could controllably activate the enhancers to compare cohesin recruitment and 3D genome architecture in the inactive versus active state, in a well characterised locus.

We subsequently used synthetic knock-out mouse models in which either all, or all but one of the enhancers in the alpha globin super-enhancer were removed, but with promoter and CTCF sites kept intact, to investigate how cohesin recruitment and the domain structure changed. Interestingly, we show that in WT erythroid cells there was approximately six times more RAD21 within the alpha globin sub-TAD than outside the region, whereas in the SE KO and the R2-only model this ratio was reduced to approximately 1:1. This striking result suggests that the presence of the enhancers influences the overall recruitment of cohesin between the characterised boundary elements known to form the edges of this sub-TAD. However, it is curious that the R2-only model and the SE KO show a very similar ratio of RAD21 recruitment. If the amount of cohesin recruitment scaled linearly with the number of enhancers it would be expected that the ratio for R2-only, with 4 enhancers removed would be a fifth of the WT, and hence still show a detectable enrichment. However, it has previously been shown that the expression of the alpha globin genes is reduced to 10% of WT levels in the R2-only model^33^ supportive of synergy between the enhancer elements which influence each other within the context of the super-enhancer and potentially explaining the inability to detect R2 dependent cohesin enrichment in the presence of the other remaining active elements such as promoters in the edited locus.

Next, we studied the strongest of the alpha globin enhancers inserted in a “neutral” region of the mouse X chromosome to investigate if this single enhancer was sufficient to recruit cohesin and form a chromatin domain. The insertion of just this short 325 bp sequence containing the R2 enhancer was surprisingly able to initiate substantial changes in both the regulatory landscape and transcription over a large region (100–180 kb). When we investigated the 3D architecture following the insertion, we observed the formation of a chromatin interaction domain across the region which in combination with the appearance of new ChIP-seq peaks of cohesin across this domain supports the model of loop extrusion as a mechanism for its formation. While the insertion site of the enhancer itself was seen to be surrounded by increased RAD21 signal, the other cohesin peaks that appeared were associated with new enhancer-like objects that appeared in the locus only upon the insertion of the R2 enhancer. As ChIP-seq measures only the steady state chromatin association of the target not its dynamics or order of recruitment, it is impossible to distinguish between a model where the active R2 element alone stimulates the local recruitment or engagement of cohesin which then translocates and stalls at these new distal sites, and a model where the activity of R2 synergises with cryptic transcription factor binding sites in the locus to cause their activation and then all elements act to stimulate cohesin recruitment. However, as the sequences of the novel ATAC-seq peaks is the same in edited and unedited cells, it is clearly the presence of the R2 enhancer element that acts as the master regulator of increased cohesin recruitment and chromatin activity at the edited locus.

It has been estimated that a high proportion of the genome carries regulatory potential^53^, it is therefore perhaps not surprising that it is challenging, or potentially impossible to find a truly “neutral” region of the genome. Whilst other studies, such as the recent one by Rinzema et al.^24^ use a random integration strategy to insert enhancers into the genome to test their ability to recruit cohesin, the rationale for our approach was the ability to control the genomic context of the insertion in order to mitigate potential confounding factors. While we took considerable care to try and define a workable neutral region in which to perform our experiments, it is clear that bringing new strong regulatory elements to a region can synergistically induce activity in sequences that are incapable of independently supporting high levels of activity or creating regulatory domains (Fig. 3A–C). A retrospective analysis of our data suggests that such potentially activatable regions may be identifiable by low levels of H3K4me1 (Fig. S10), though this will require further investigation to determine how predictive this may be to identify activatable cryptic elements.

One of our more puzzling observations is the tendency for the R2 element, once inserted into chrX, to exhibit high levels of H3K4me3 and unidirectional transcription, which would ordinarily be indicative of a promoter element. However, it still remains to be understood how genomic context can act to dramatically alter the behaviour of such a well characterised enhancer. However, this dramatic change in behaviour does add further weight to the idea that regulatory elements should be considered to have a spectrum of activity rather than being classified into distinct dichotomous groups of either promoters or enhancers^54–56^. However, peaks observed in ChIP-seq ultimately reflect the steady-state enrichment of cohesin at sites across the genome, therefore represent the aggregate signal from loading, translocation or stalling of cohesin on chromatin through the effect of boundary elements such as CTCF. Current technologies such as ChIP-seq cannot assess protein dynamics on chromatin so it is still not possible to pinpoint the exact sites where cohesin is initially recruited compared to where it translocates and stalls, highlighting an important technical challenge for the field in the future.

In parallel to this study our lab is currently working on the behaviour of the two different cohesin isoforms: STAG1 and STAG2 in cohesin recruitment^32^. In brief, we observe both STAG1 and STAG2 at all elements of the alpha globin super-enhancer but only in erythroid cells. We noted that the peaks of STAG2 are more prominent than STAG1, which aligns with the other work in the field reporting that STAG2 is the isoform most likely responsible for the formation of enhancer-promoter interactions required for the activation of specific gene transcriptional activation programmes^57–59^. In the work presented in this present study, cohesin recruitment does not appear to be related to the presence of CTCF at the 5 alpha-globin gene enhancers, we hope that our work on the STAG isoforms may help to further explain the mechanism of the CTCF independent cohesin recruitment at these enhancer elements.

The results presented in this study bring us a step closer to understanding how 3D genome architecture can be directed via the recruitment of loop extrusion processes by active regulatory elements in a cell type specific manner. These results suggest that regulatory elements, in addition to stimulating transcription from promoter elements, have co-evolved an important function to allow them to stimulate the processes, which in concert with functional boundary elements, bring them in close proximity to the promoters they evolved to activate.

## Materials and methods

A full list of cell models used in this work are supplied in Table S4. Experimental procedures were in accordance with the European Union Directive 2010/63/EU and/or the UK Animals (Scientific Procedures) Act (1986) and protocols were approved through the Oxford University Local Ethical Review process.

### CD34 + HSPC isolation and differentiation

CD34 + human stem and progenitor cells (HSPCs) were isolated from the three donors (1, 2 and 3), expanded and differentiated up until day 10 as previously descibed^60^. The donor material was sourced from the Oxford Biobank (https://www.oxfordbiobank.org.uk/) which holds informed consent for the experimental techniques performed and data generated in this project. The project design was assessed and accepted by the Oxford Biobank Steering committee.

### Mouse embryonic stem cell culture and genome engineering

E14TG2a.IV mESCs were maintained by standard published methods^61,62^. CRISPR/Cas9 mediated HDR strategies were used to produced genetically modified cell lines. mESCs were co-transfected with guide RNA and HDR vectors using Lipofectamine LTX reagent (ThermoFisher) according to manufacturer’s instructions. Sequences for guide RNA and HDR vectors are supplied in Table S2.

### Isolation of erythroid cells derived from adult mouse spleen

We generated single-cell preparations of erythroid cells by gently dissociating cells from the spleens of 6–9-month-old female mice (F1 crosses between C57BL/6 and CBA/J and pure C57BL/6) treated with phenylhydrazine (40 mg/g body weight per dose, with three doses given 12 h apart; mice were killed on day 5). Mice were maintained in specific pathogen–free facilities at the Biomedical Services Unit of Oxford University. All mouse work was performed in accordance with UK Home office regulations, under the appropriate animal licenses. Protocols were approved through the Oxford University Local Ethical Review process. Experimental procedures were performed in accordance with European Union Directive 2010/63/EU and/or the UK Animals (Scientific Procedures) Act, 1986. Phenylhydrazine causes hemolytic anemia and marked erythroid expansion in the spleen so that 80% or more of cells are erythroid cells (defined as CD71 + ter119+). The spleens were harvested and mechanically disaggregated to cell suspension which was passed through a 40-µm CellTrix strainer (Sysmex) to remove clumps. For ter119 + cell selection, we stained cells with ter119-phycoerythrin and positively selected using anti-phycoerythrin MACS beads (Miltenyi Biotec). Wildtype (C57BL/6) animals were obtained from the Biomedical Services at the University of Oxford, purchased from approved providers. The mice are sacrificed following Animals (Scientific Procedures) Act 1986 Schedule 1 Standard Method of Humane Killing for adult mice (such as dislocation of the neck followed by confirmation of death). This study is reported in accordance with ARRIVE guidelines. There was a minimal reliance on mouse material in this study and it was only restricted to wildtype adult animals, taking into account ARRIVE guidelines, as detailed above.

### Embryoid body differentiation and erythroid population isolation

EB differentiation and CD71 + erythroid population isolation was performed following the previously published protocol^38^, aliquots were used fresh or frozen at -80ºC depending on the downstream protocol.

### ATAC-seq

ATAC-seq was performed on 75,000 cells from target populations as previously described^34,63^. ATAC-seq libraries were sequenced with a high-Output v2 75 cycle kits on the Illumina NextSeq platform.

### ChIP-seq

ChIP-seq was performed on aliquots of 3-10 × 10^6^ CD71 + cells. For calibrated ChIP-seq, a 1% or 4% spike-in of WT HEK293 cells were added prior to sonication. Cells were double cross-linked using disuccinimidyl glutarate (DSG, Sigma) and 1% formaldehyde (Sigma) for a total fixation time of 1 h. Fixed chromatin samples were fragmented using the Covaris sonicator (ME220) for 10 min (75 power, 1000 cycles per burst, 25% duty factor) at 4 °C. 50µL per sample was removed as an input control. Sonicated samples were pre-cleared to remove background signal through incubation with a 1:1 mix of Protein A/G Dynabeads (InVitrogen). Antibody was added at a concentration of 1 µg/µL per sample and immunoprecipitated at 4 °C overnight, a full list of antibodies used is supplied in Table S3. Immunoprecipitated samples were incubated with a 1:1 mix of Protein A/G Dynabeads for 5 h at 4 °C. Beads were then washed 4x in RIPA buffer on a magnetic stand, followed by 1x wash with TE (Sigma Aldridge) + 50mM NaCl (ThermoFisher). Chromatin was eluted from the beads using elution buffer and incubated for 30 min at 65 °C with shaking. Input samples were diluted 1:1 with elution buffer. Samples and input controls were incubated at 65 °C overnight for de-crosslinking, before RNase (Roche) and proteinase K (BioLabs) treatment. DNA fragments were purified using the Zymo ChIP DNA Clean & Concentrator kit (Zymo Research) and eluted in water. DNA concentration was quantified using the Qubit dsDNA HS assay (Invitrogen) as per manufacturers protocol. To assess sonication efficiency the D1000 Tapestation (Agilent) assay was performed on input sample. An approximately equal mass of input and IP DNA was used for indexing (0.5 ng – 1 µg). NEBNext Ultra II DNA Library Prep Kit (New England Biolabs) was used to prepare indexed sequencing libraries following the manufacturer supplied protocol. PCR amplification was performed for 7–11 cycles (depending on input DNA concentration) using NEBNext Mulitplex Oligos (New England Biolabs). Indexed sample concentration was quantified using the KAPA Library Quantification Complete Kit (Universal)(Roche). Samples were pooled as a 4 nM library and sequenced with a high-Output v2 75 cycle kits on the Illumina NextSeq platform.

### Next-generation capture-C

Next-generation Capture-C was performed as previously described^64^ on 5 × 10^6^ mouse CD71 + cells derived from three separate differentiation experiments. Custom biotinylated DNA oligonucleotides used in Capture-C experiments are supplied in Table S1. Capture-C libraries were sequenced on the Illumina Nextseq platform using a 300-cycle paired-end kit (NextSeq 500/550 Mid Output Kit v2.5). To produce sufficient sequencing depth > 1 × 10^6^ 150 bp paired-end reads were generated per viewpoint, per sample in multiplexed library.

### Tiled-C

Tiled-C experiments were performed on mESC and erythroid cells previously described^1,33^.

### RNA extraction

Total RNA was isolated from 1 to 5 × 10^6^ CD71 + from cultures of embryoid bodies. Cells were lysed in TRI reagent (Sigma-Aldrich) using a Direct-zol RNA MiniPrep kit (Zymo Research). DNase I treatment was performed on the column as recommended in the manufacturer’s instructions, with an increased incubation of 30 min at room temperature (rather than the recommended 15 min). RNA was eluted in DNase/RNase-free Water and degradation was assessed using RNA Screentape Analysis (Tapestation, Agilent Technologies) which determined the RNA integrity number (RIN) score of the sample. RNA was quantified either by Qubit RNA Broad-Range Assay (Invitrogen, ThermoFisher). RNA samples were stored at − 80 °C prior to RNA-seq.

### Poly(A)+/− RNA-seq

1–2 µg of total RNA was depleted of rRNA and globin mRNA using the Globin-Zero Gold rRNA Removal Kit (Illumina) according to the manufacturer’s instructions. Samples were purified at -80 °C overnight by ethanol precipitation, centrifuged for 1 h at 12,000 g, washed twice with 70% ethanol, and resuspended in RNase-free water. RNA Screentape Analysis (Tapestation, Agilent Technologies) for quality control to ensure sufficient depletion and integrity of RNA samples. For mRNA enrichment: Poly(A) + RNA was isolated, strand-specific cDNA synthesised, and the resulting libraries prepared for Illumina sequencing using the NEBNext Poly(A) mRNA Magnetic Isolation Module (New England Biolabs) and the NEBNext Ultra II Directional RNA Library Prep Kit for Illumina (New England Biolabs) following the manufacturer’s instructions. The poly(A)- RNA fraction was retained by storing the supernatant and all subsequent washes of the magnetic Oligo dT Beads bound with mRNA at − 80 °C.

Poly(A)- samples were purified using Agencourt RNAClean XP beads (Beckman Coulter) and eluted in RNase free water. To remove any contaminating poly(A) + RNA, an additional poly(A) + selection was performed using the magnetic Oligo dT Beads from the NEBNext Poly(A) mRNA Magnetic Isolation Module following the manufacturer’s instructions. Poly(A)- RNA samples were purified using Agencourt RNAClean XP beads and eluted in the First Strand Synthesis Reaction Buffer and Random Primer Mix (2X) from the NEBNext Ultra II Directional RNA Library Prep Kit for Illumina. Fragmentation of RNA, strand-specific cDNA synthesis, and library preparation for Illumina sequencing were all performed using the NEBNext Ultra II Directional RNA Library Prep Kit for Illumina according to the manufacturer’s instructions.

For both Poly(A) + and Poly(A)- samples: purification of the double-stranded cDNA, the adaptor ligation reaction, and the PCR reaction of adaptor ligated DNA were performed using Agencourt AMPure XP beads. Library profiles were assessed by visualisation on a D1000 tape on a Tapestation (Agilent Technologies) and quantified using a universal library quantification kit (KAPA Biosystems, Roche: 07960140001). Poly(A) + and poly(A)- RNA-seq libraries were sequenced on the Illumina Nextseq platform using a 75-cycle paired-end kit (NextSeq 500/550 High Output Kit v2.5: 20024906).

### Data analysis

#### ATAC-seq and ChIP-seq

For publicly available data, raw data was downloaded in SRA format from the relevant GEO repositories. Using the SRA Toolkit^65^ ‘fastq-dump’ function, fastq files were extracted. ATAC-seq and ChIP-seq data in fastq format were analysed using the CATCH-UP pipeline^66^. In brief, single- or paired-end reads were aligned to the target genome using Bowtie2^67^. Samtools^68^ was used to remove duplicates and index bamfiles. A coverage track of the aligned reads was generated using deepTools^69^ ‘bamCoverage’, where coverage was calculated for a single base pair window and replicates were normalised to Reads Per Kilobase per Million mapped reads (RPKM) using flags ‘-bs 1 --normalizeUsing RPKM’. The output coverage files in bigWig format were visualised using the UCSC Genome Browser^70^.

#### Calibrated ChIP-seq

The pipeline for the analysis of calibrated ChIP-seq data was adapted from the scripts used in Fursova et al.^71^, and is available in the UpStreamPipeline GitHub repository. In brief: (1) whole genome fasta files for the target and spike-in species were concatenated and used to build Bowtie2 index files; (2) paired-end reads were then aligned to the concatenated genome using Bowtie2^67^; (3) reads were separated and counted based on alignment to target or spike-in genome and a down-sampling factor was calculated based on the total number of spike-in reads per sample as follows:

1 $${\text{down - sampling factor = a}} \times \frac{1}{{{\text{total}}\:{\text{reads}}\:{\text{spike}} - {\text{in}}\:{\text{ChIP}}}} \times \frac{{{\text{total}}\;{\text{reads}}\:{\text{spike}} - {\text{in}}\:{\text{Input}}}}{{{\text{total}}\:{\text{reads}}\:{\text{target}}\:{\text{Input}}}}$$

where α = coefficient for bulk normalisation of samples within a single experiment to enable the value of the largest down-sampling factor to equal 1^71^. (4) using the calculated down-sampling factor, reads for each ChIP sample were randomly subsampled using Sambamba^72^. (5) Input samples were used to correct the ratio of spike-in to target read counts across the biological replicates to account for minor variations in the mixing of spike-in cells, (6) sub-sampled bamfiles were then processed according to the standard ChIP-seq analysis method described above.

#### Next-generation capture-C

Capture-C data were analysed as previously described^64^ aligning to the mouse mm9 reference genome. CaptureCompare was used to generate comparisons of the Capture-C interaction profiles (https://github.com/djdownes/CaptureCompare). Briefly, unique interactions were normalised to cis interactions, averaged (*n* = 3), and the difference between the means calculated. For visualisation, means and standard deviations were binned into 150 bp bins and smoothed with a sliding window of 3 kb, and plotted using ggPlot in R.

#### Tiled-C

Tiled-C data was analysed using the tCaptureC workflow available in the UpStreamPipeline GitHub repository. This uses Hi-C Pro^73^, with DpnII digestion of the genome using the built-in ‘digest-genome’ utility. Capture target was specified using an input BED file containing region coordinates, min/max fragment sizes were specified in the config file as 20 and 100,000 respectively, and ICE normalisation implemented. All other parameters applied were set at default. HiCPlotter^43^ was used to visualise the interaction matrices.

#### Custom genome builder

To correctly align reads to the genetically engineered R2-insertion cell lines a custom genome was built. In brief, this pipeline takes a fasta file containing the insert sequence and a BED file of the coordinates of the edited region, and bioinformatically cuts-and-pastes together a custom genome. Samtools^68^ was used to index the custom genome fasta file, and Bowtie2^67^ used to create new indices.

#### Poly(A)+/− RNA-seq

RNA-seq reads were aligned to the genome (mm39) using STAR^74^ with --outFilterMultimapNmax value set to 2. Alignment reads were filtered for proper pairs, sorted, and PCR duplicates removed using Samtools^68^. Biological replicates were normalised to RPKM in a strand-specific manner using deepTools^69^ bamCoverage and the flag --filterRNAstrand. Biological replicates were merged and converted into bigWig file format for visualisation in the UCSC genome browser.

#### Allelic skew, meta-analysis and motif analysis

Phased genomes were variant called on 10x sequencing data using longranger (v2.2.2) and the Genome Analysis Toolkit (GATK)^75^. From the phased genomes ‘bcftools consensus’ was applied to create personalised genomes. To remove alignment biases, all datasets, for each donor, were aligned to the hg38 reference genome, in addition to the two personalised genomes. ATAC-seq read counts for skew analysis were obtained using the pysam (v0.20.0) ‘pileup’ function and matched by haplotype. Alignment bias discrepancies were removed by setting a read count of 0 at variant locations for both alleles when necessary. As a result of having phased Variant Call Formats (VCFs) we have the ability to resolve loci using others in phase loci. Variants were identified when found on all haplotypes with a positive, or on all haplotypes with a negative skew. Only variants within the peak called regions were analysed.

51 regions across the genome were selected based on skew analysis in which the three donors were a mix of homozygous-ref, heterozygous and homozygous-alt where the alternative haplotype was shown to exhibit differential signal (or skew) in ATAC-seq, with stringent p-value threshold of *p* < 0.0001. To calculate the RAD21 coverage across the 51 regions, the BedTools^76^ ‘multicov’ function was used, with a bedfile of the 51 regions and bamfiles of RPKM normalised RAD21 ChIP-seq data from the three donors. Data was visualised using the Python (3.11.3) package Seaborn (0.12.2), with p-values calculated using the Scipy (1.10.1) paired T-test.

We run our phased allelic read-counts through WASP^77^. For the variants we input this produces a p-value for the null hypothesis that there is no allelic skew.

For Promoters, Enhancers, and CTCF regions we analyse SNVs which have consistent allelic skew in RAD21 and ATAC (Enhancers/Promoters) or CTCF ChIP (CTCF). These are the SNVs which make the bottom-left and top-right quadrants of Fig.S6. For each SNV we analyse the associated peak region in two ways: The first analysis takes the full sequence found in the peak-call region and the second analysis takes the 20 bps upstream and downstream of the SNV. For each SNV sequence (whether full peak-call region or local 40 bps), we obtain the up-skewed sequence from the up-skewed allele and the down-skewed sequence from the down-skewed allele using the phased genomes. These are built into fasta files which are then processed using HOMER^78^ as follows:findMotifs.pl fasta.up.Enhancer.skewed.fa fasta MotifsEnhancer/ -fastaBg fasta.down.Enhancer.skewed.fa.

The output from this command is used to build the excel table (Supplementary Material Table 5). The detected motifs have a human friendly label based upon HOMER performing a database search, motif-sequence, p-values, q-value, then frequency counts detailing how often the motif-sequence is found in the up-skewed alleles vs. the down-skewed alleles.

#### RAD21 coverage analysis for enhancer KO models

Two regions: (1) chr11: 32,188,452–32,249,902 and (2) chr11: 32,323,049–32,384,895 were provided in bed format along with bamfiles of RAD21 ChIP-seq for WT, R2-only and SE KO models, and to calculate coverage scores for each replicate using the BedTools^76^ ‘multicov’ function. Data was visualised using the Python (v3.11.3) package Seaborn (v0.12.2).

#### CTCF motif analysis

CTCF ChIP-seq data was peak called using Lanceotron^79^ with default settings and fasta sequence for each peak extracted using the Bedtools ‘getfasta’ utility^76^. The MEME frequency matrix for CTCF (CTCF_MA0139.1.meme) was downloaded from JASPAR^80,81^. Using the FIMO (Find Individual Motif Occurrences)^82^ tool from The MEME suite^80^, occurrences of MA0139.1 were determined within the CTCF peak calls using default parameters. Peaks with identified CTCF motifs were annotated with the orientation.

#### CRE classification using REgulamentary^35^

ATAC-seq and ChIP-seq data for CTCF, H3K4me1, H3K4me3 and H3K27ac is pre-processed using CATCH-UP^66^ and then input into the REgulamentary pipeline. In brief, this tool uses a rule-based ranking approach to classify cis-regulatory elements in a cell-type specific manner based on their histone mark signatures. For a detailed methodology refer to the GitHub repository linked in Code Availability and the original publication.

## Electronic supplementary material

Below is the link to the electronic supplementary material.

Supplementary Material 1

Supplementary Material 2

## Data Availability

ATAC-seq, ChIP-seq, RNA-seq, Capture-C and Tiled-C data generated for this study have been deposited in the Gene Expression Omnibus (GEO) under accession code GSE244929. Previously published ChIP-seq and ATAC-seq data that were reanalysed here are available under the following accession codes: GSE57092, GSE30203, GSE30203, GSE36028 and GSE97871. Previously published Tiled-C data that were reanalysed here are available under the accession code GSE137477. Previously published data from the R2 only and DSE models that were reanalysed here are available under the accession code GSE220463. UCSC session for Oxford Biobank donors 1,2,3 ChIP-seq (H3K4me1, H3K4me3, H3K27ac, RAD21, CTCF) and ATAC-seq: https://genome.ucsc.edu/s/egeorgia/2023-01 Oxford Biobank genomics %28hg38%29 updated UCSC session for R2-insertion vs. WT in artificial locus ChIP-seq (H3K4me1, H3K4me3, H3K27ac, RAD21, CTCF), RNA-seq, and ATAC-seq: https://genome.ucsc.edu/s/egeorgia/artificial_locus_genomics All other data supporting the findings of this study are available from the corresponding author on reasonable request.
