# Supplementary material for: Active regulatory elements recruit cohesin to establish cell specific chromatin domains
Source: Sci Rep. 2025 Apr 6;15:11780. doi: 10.1038/s41598-025-96248-4 (PMC11973168; doi:10.1038/s41598-025-96248-4)
Supplement: Supplementary file 2 — Supplementary Material 2 [file 41598_2025_96248_MOESM2_ESM.docx]

**Supplementary Material**

Emily Georgiades^1†^, Caroline L. Harrold^1†^, Nigel Roberts^1^, Mira Kassouf^1^, Simone G. Riva^2^, Edward Sanders^2^, Damien Downes^1^, Helena S. Francis^1^, Joseph Blayney^1^, A. Marieke Oudelaar^3^, Thomas A. Milne^1^, Douglas R. Higgs^1,4^* & Jim Hughes^1,2^*

^1^ MRC Molecular Haematology Unit, Weatherall Institute of Molecular Medicine, Radcliffe Department of Medicine, University of Oxford, Oxford, UK

^2^ MRC WIMM Centre for Computational Biology, Weatherall Institute of Molecular Medicine, Radcliffe Department of Medicine, University of Oxford, Oxford, UK

^3^ Max Planck Institute for Multidisciplinary Sciences, Am Fassberg 11, 37077 Göttingen, Germany

^4^ Chinese Academy of Medical Sciences Oxford Institute

^†^ Contributed equally

* Corresponding authors; email: jim.hughes@imm.ox.ac.uk and doug.higgs@imm.ox.ac.uk

**Fig. S1: Cis-regulatory element classification of open chromatin sites in Donor 1 using REgulamentary.**

ATAC peaks with a peak score > 0.95 for Donor 1 were used as input to REgulamentary (see Materials and Methods), along with ChIP-seq data for H3K4me1, H3K4me3, H3K27ac and CTCF, in order to classify the open chromatin sites into the corresponding cis-regulatory element classes. Heatmaps show the sorted coverage scores ±2kb from the peak center separated by class, summary line plots for the average peak shape are shown above each column.

**Fig. S2: Cis-regulatory element classification of open chromatin sites in Donor 2 using REgulamentary.**

ATAC peaks with a peak score > 0.95 for Donor 2 were used as input to REgulamentary (see Materials and Methods), along with ChIP-seq data for H3K4me1, H3K4me3, H3K27ac and CTCF, in order to classify the open chromatin sites into the corresponding cis-regulatory element classes. Heatmaps show the sorted coverage scores ±2kb from the peak centre separated by class, summary line plots for the average peak shape are shown above each column.

**Fig. S3: Cis-regulatory element classification of open chromatin sites in Donor 3 using REgulamentary.**

ATAC peaks with a peak score > 0.95 for Donor 3 were used as input to REgulamentary (see Materials and Methods), along with ChIP-seq data for H3K4me1, H3K4me3, H3K27ac and CTCF, in order to classify the open chromatin sites into the corresponding cis-regulatory element classes. Heatmaps show the sorted coverage scores ±2kb from the peak center separated by class, summary line plots for the average peak shape are shown above each column.

**Fig. S4: Natural variation example at chr6:79,507,436-79,722,535.**
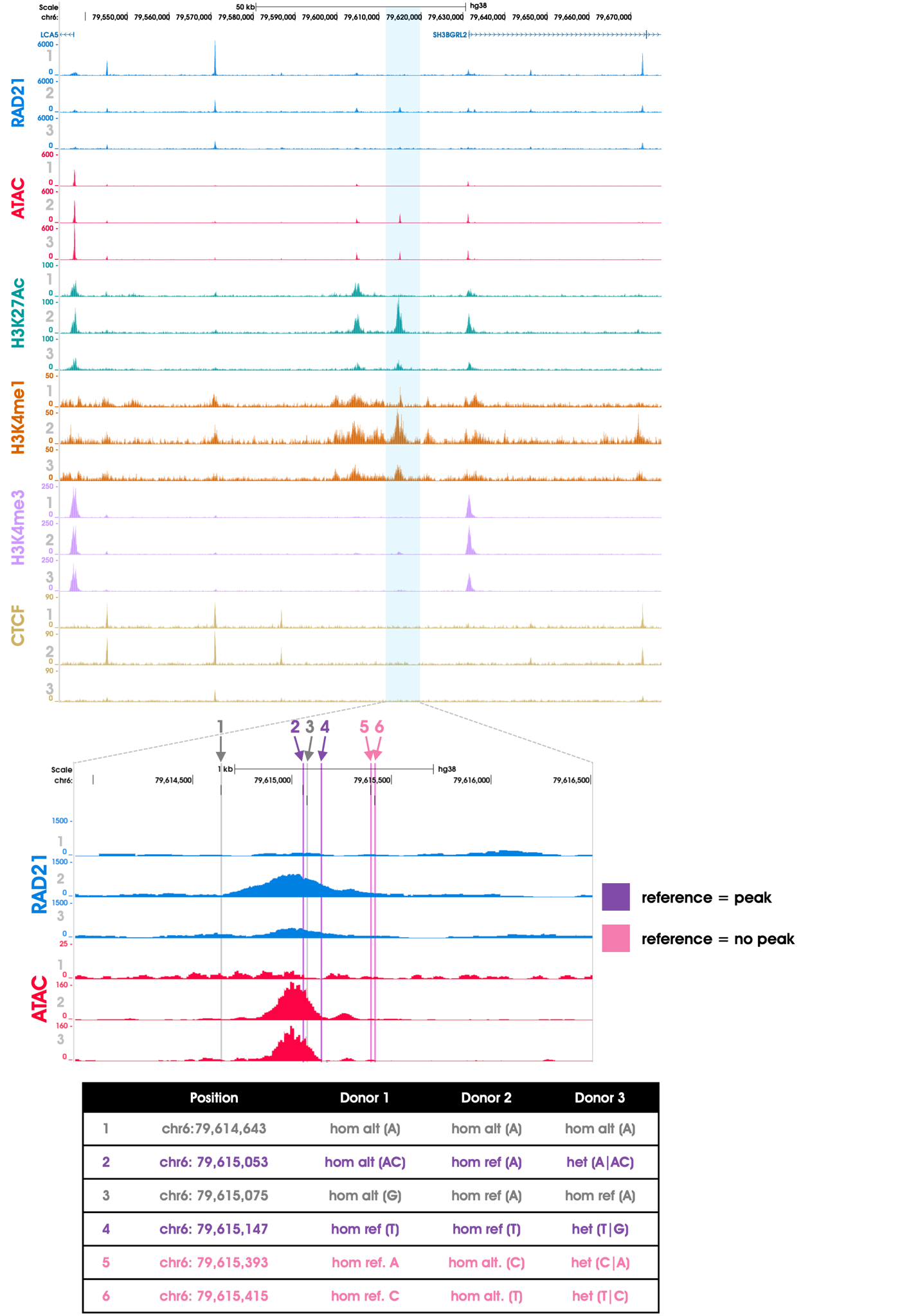


1

Region shown is chr6:79,507,436-79,722,535 (hg38). Numbers 1, 2, 3 indicate the anonymized donor identifiers. ChIP-seq for RAD21, CTCF and histone marks (H3K4me1, H3K4me3, H3K27ac) are shown for each donor in the top panel along with the open chromatin signal (ATAC-seq). The region highlighted in blue is shown in detail below. Here the difference in signal across the donors can be clearly seen: donor 1 is homozygous and displays no peaks in ATAC-seq or RAD21, donor 2 is homozygous (alternative allele to donor 1) and displays a positive ATAC-seq signal and the strongest RAD21 signal, donor 3 is heterozygous with a peak in ATAC-seq and moderate RAD21 signal. The pink (refernce = peak) and purple (reference = no peak) lines indicate the SNP we have identified as potentially causal for these differences across the donors.

**Fig. S5: Natural variation example at chr1:154,364,808-154,566,107.**


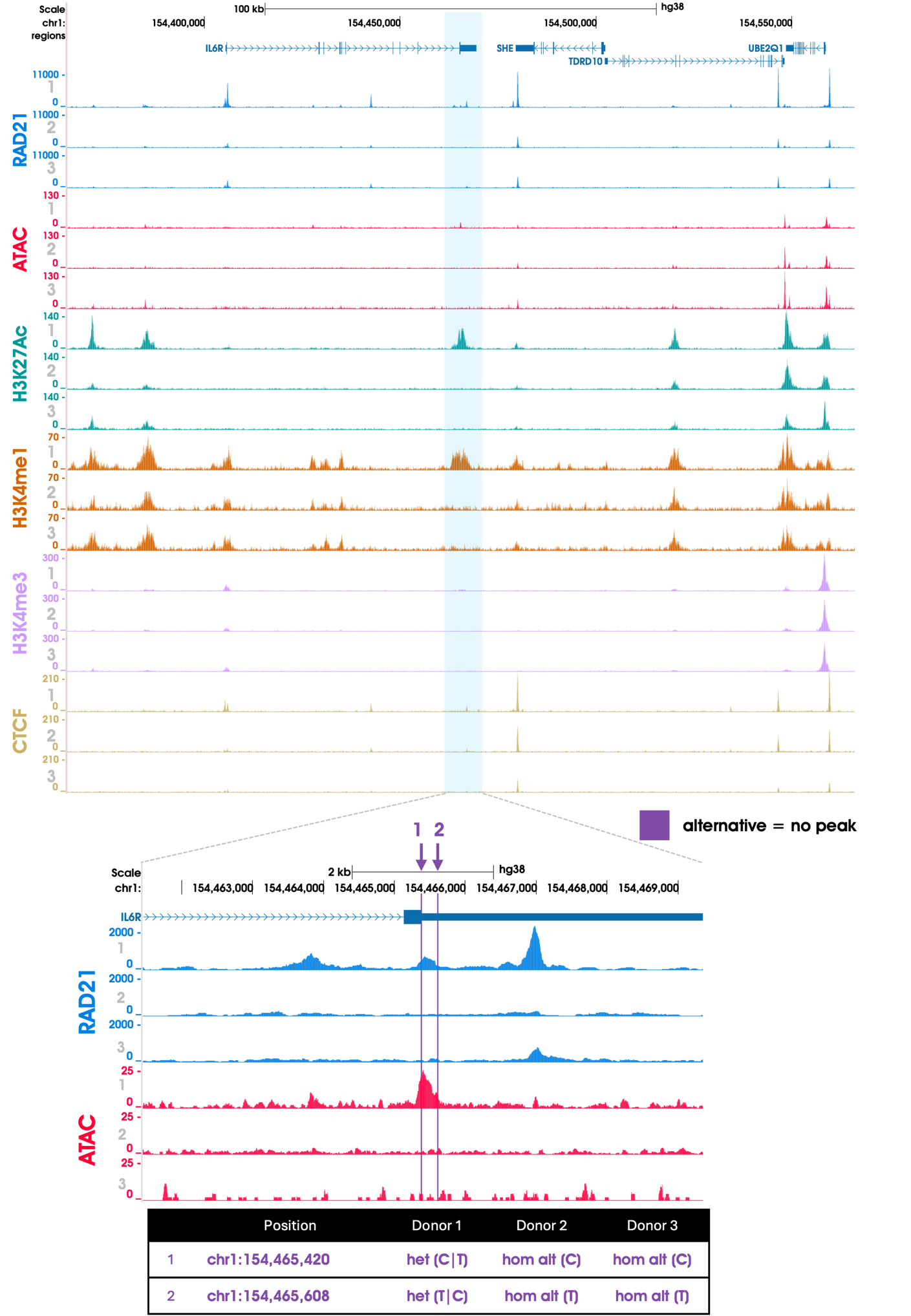


Region shown is chr1:154,364,808-154,566,107 (hg38). Numbers 1, 2, 3 indicate the anonymized donor identifiers. ChIP-seq for RAD21, CTCF and histone marks (H3K4me1, H3K4me3, H3K27ac) are shown for each donor in the top panel along with the open chromatin signal (ATAC-seq). The region highlighted in blue is shown in detail below. Here the difference in signal across the donors can be clearly seen: donor 1 is heterozygous and displays a positive ATAC-seq signal and a peak of RAD21, donor 2 and donor 3 are is homozygous and display no ATAC-seq or RAD21 signal. The purple (alternative = no peak) lines indicate the SNPs we have identified as potentially causal for these differences across the donors.

**
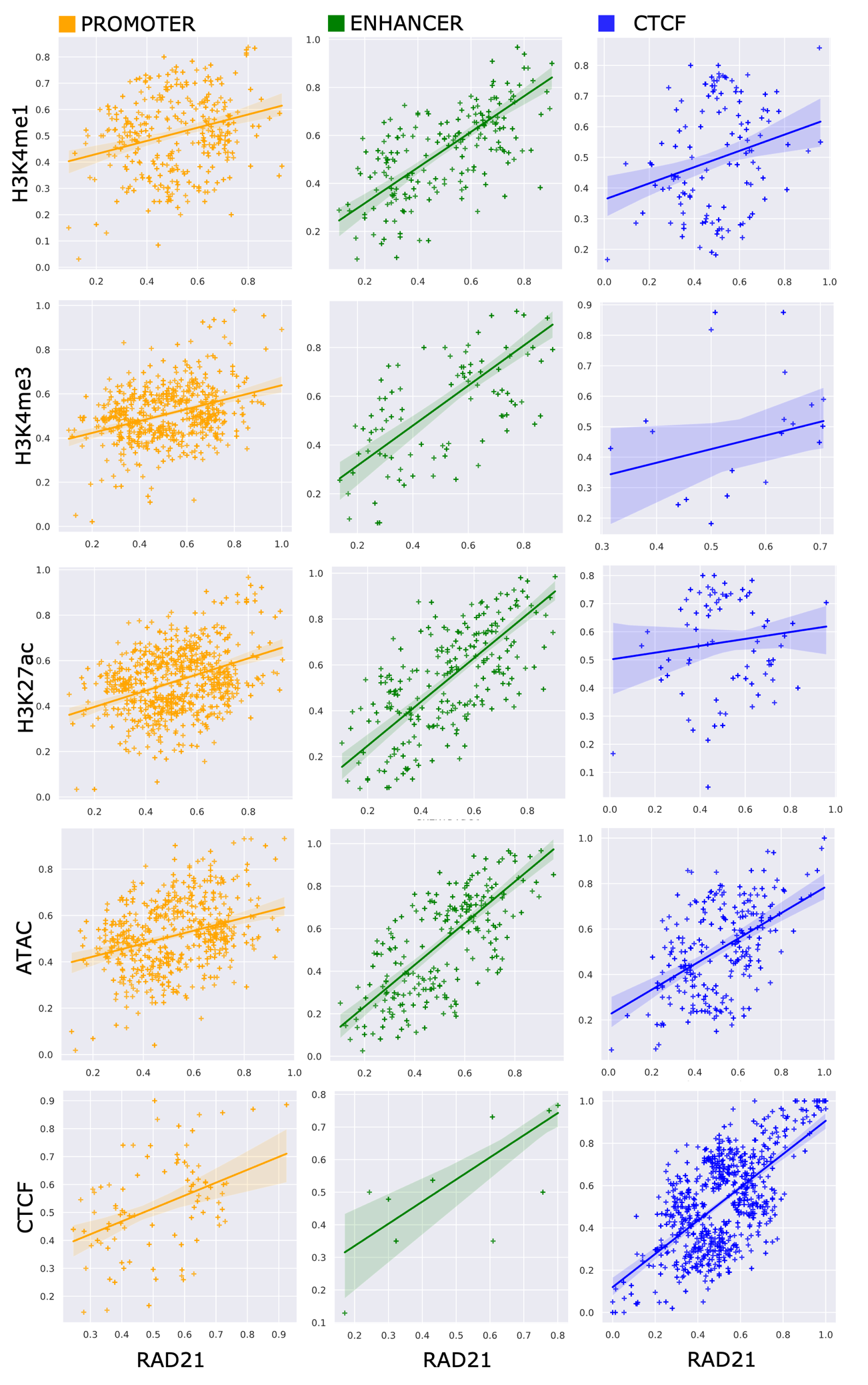
Fig. S6: Genome-wide allelic skew analysis.**

Scatter plots show the skew across RAD21 sites (x-axis) against skew in each of: H3K4me1, H3K4me3, H3K27ac, ATAC and CTCF (y-axis). Plots are coloured based on the classification of sites as either promoter (green), enhancer (yellow), or CTCF only (blue). For full details of analysis, refer to Material and Methods.

**Fig. S7: Identification of an activatable region devoid of active histone marks, open chromatin and CTCF binding.**

Characterisation of the chromatin environment across chromosome X was performed and the region chrX:11,018,725-11,521,742 (mm39) was identified to fit all the criteria to enable targeting of R2 to this site. All data is RPKM normalised. Publicly available data sources are listed in Supplementary Table 4, all other data is available to download from GSE244929.

**Fig. S8: There is no underlying 3D structure within the selected chromosome X region.**

Next-generation (NG) Capture-C was performed from the six viewpoints shown (LP1-LP6) and highlighted in blue. Data from WT mESC is shown in blue and WT erythroid cells in purple. RPKM normalised ATAC-seq (dark pink) and CTCF (gold) data in WT erythroid cells are shown for reference.

**Fig. S9: Comparison of the ATAC-seq reads originating from the R2 enhancer in WT versus the R2-insertion erythroid cells.**


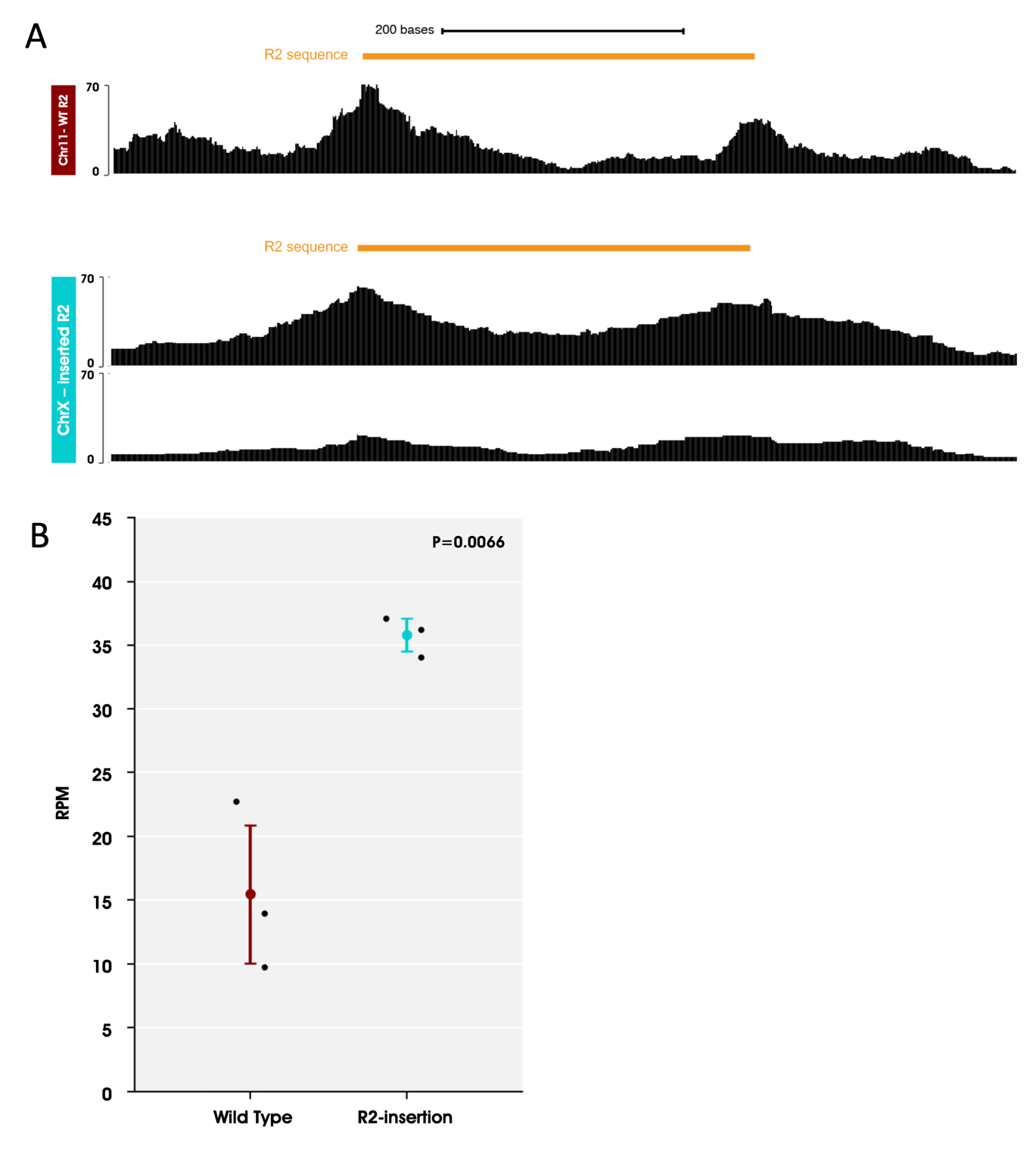


(A) Profiles show paired-end reads from ATAC-seq in R2-insertion erythroid cells that spanned the junctions of R2 in chr11 and chrX. Paired-end reads that originated from the 325 bp sequence of R2 (orange bar) were discarded. Profiles from representative clones; two clones appeared to have a similar level of chromatin accessibility to chr11-R2 junctions, two clones appeared to have a reduced level of chromatin accessibility. (B) Reads that overlapped with the 325 bp R2 sequence normalised to reads per million (RPM) for ATAC-seq performed on WT and R2-insertion erythroid cells. Mean and standard deviations for each model are shown, each point represents a biological replicate/independently targeted clone. The mean of R2 reads from ATAC-seq in the R2-inseryion erythroid cells significantly differ from that of WT, p-value (p=0.0066) shown was calculated using a paired two-tailed student’s T-test.

**Fig. S10: Characterisation of the histone landscape in WT cells.**

The histone modifications H3K4me1 (orange), H3K4me3 (lilac), H3K27ac (turquoise) are compared across three unedited WT mouse cell types: ESCs (no. 1), primary erythroid cells derived from spleen (no. 2) and erythroid cells derived from embryoid body cultures (no. 3), across two loci: (A) the chromosome X region (chrX:11,126,444-11,453,282) that was selected for targeting, and (B) the alpha globin locus (chr11:32,123,214-32,270,752). The location of the endogenous R2 enhancer in the alpha globin locus is highlighted in blue for reference. Data sources for publicly available data sets are detailed in Supplementary Table 4, all other data was generated by the authors and is available in GSE244929.

**Fig. S11: Characterisation of the histone landscape in WT and R2-insertion embryoid body derived erythroid cells.**

The histone modifications are shown as follows: H3K4me1 (orange), H3K4me3 (lilac) and H3K27ac (turquoise), across the region chrX:11,126,444-11,453,282. The R2-insertion site is indicated above the tracks by a purple rectangle. All tracks are RPKM normalised. Raw and processed data are available to download from GSE244929.

**Fig. S12: Classification of enhancers and promoters by H3K4me1/H3K4me3 signatures.**

**
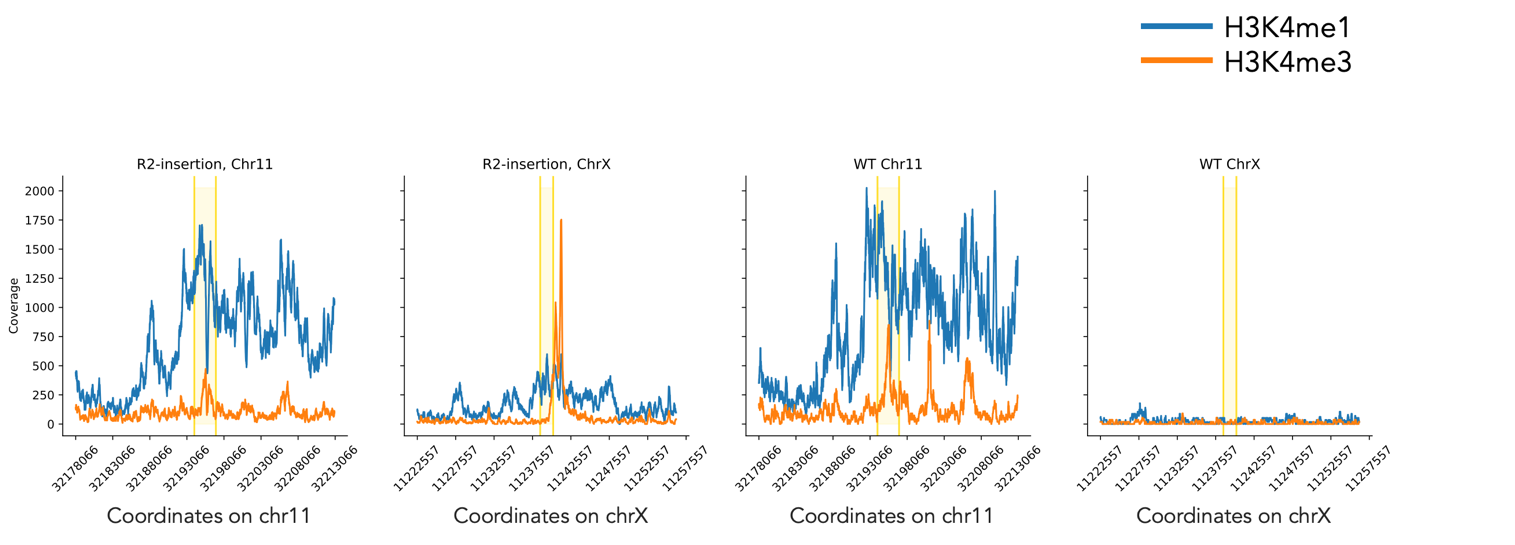
**

Panels show the H3K4me1 (blue) and H3K4me3 (orange) signal at the native (chr11) and insertion site (chrX) in WT and the edited (R2-insertion) embryoid body cells. The exact location of the R2 enhancer and the insertion site are highlighted in yellow. Coordinates of the up and downstream region are annotated on the x-axis and coverage of the histone marks (RPKM normalised) is shown on the y-axis.

**Table S1: Capture oligonucleotides were designed to six viewpoints (LP1-LP6) across the chromosome X locus.**

| **Viewpoint** | **Capture DNA oligo** | **Sequence (5’-3’)** |
| --- | --- | --- |
| LP1 | 5’ | [Btn]GATCTATATGGGATGAGATATTAGTTGGGGTTCTCCAGAGACAGGGAGCCAATAAGATGTATGTTGTTGG |
|  | 3’ | [Btn]CCCAGCCAAGAGAATGGTGTCATTCACAACTGGCTGGGTTTTCTTGTATCAATTAAGAGTCAAGATGATC |
| LP2 | 5’ | [Btn]GATCATGGAATATTTTTTCTTTTGGACTTGCTTCACTCAAATAAAAAAACTGTTCACCTCATGTTTTCCA |
|  | 3’ | [Btn]TGAATTCCCTGAAGGTAAGAACAGGAATTATCTTATCTCTAAACCAGGTATTGGGCAATTCAGGTGGATC |
| LP3 | 5’ | [Btn]GATCATTAAAGGAGACAGGAGGAAAGGATACCATCCCCGCAGCAGGGAAATGGACTGCAACAGAGCATCC |
|  | 3’ | [Btn]ATGAGGCTTTGTCCTAAGCGGCTAAGTGTCCCCGGTGTTCCTAGCAGGAATACAGGCTGGCTCATGGATC |
| LP4 | 5’ | [Btn]GATCCAAAATGCTCTGCTGAACTCAAGATAGTTCATTTCACTAAAGGCCGTGCTGAGGAGCAGGGGCAAA |
|  | 3’ | [Btn]GCTACCATGTAAGCCCCAGGACCAGCTATGTCTTGCTCCTGGAGAAGGCGGTCATCATGGCTACCTGATC |
| LP5 | 5’ | [Btn]GATCTAGTCCTCACACATTTATGAGATGGTGTCACCAAGAGCACCTTCACAAGAAGACCAATGCGATACC |
|  | 3’ | [Btn]TACTTTTCAAAGAAACTAAAAATTCTCACTACCTGCATTCATCATCCAAAATTCTCTAGAGAAACAGATC |
| LP6 | 5’ | [Btn]GATCCAAATGTTCTTAAGGTCCTGAAGTAGTTTTTAAAAATAGACTTCCGTGAAGAAAGAACTCTGGTTG |
|  | 3’ | [Btn]TATTTTAAAAGAAGAAAAGGAATAGACATTTGTGTAGGTCATGTTGCTGGAGTCTAGAAAGCCTAAGATC |

**Table S2: Sequences for guide RNA and HDR vectors.**

| **Target** | **Sequence 5’-3’** |
| --- | --- |
| LP2  guide | GGAGAGTAGTGGCCCAACTCT |
| LP2 R2-insertion  donor | CCCACAATGGCATGGCATTGCTGAATGTATCCCAGTTTTTTGGCTTTTGATGCCTATATTGTCAGGGTCATATATCCCCAACCACTACTGAGAAAAACTTCCTTGGGTCAGTGTTGGATAGCAGCAGCTCCCTTATGCTTACTTGGAGTGGTTCTAAGATTTTGTGTTTTACATTAGGTATTTTATCTATTTGCAGTTGGTTTGGGATGTTGTGTGAGACAAGGGTCCAGTTTCTTTCTTCTGCATGTACACATCTAGTCTTCAAACAGAGTTCAATGAAGAAATTGTCAATTTTCTATTGTATATACTTTTGCCATATTCATGCTGGAAATGGCTTTCTTTGCTTTTCCCAAGGTAATCTGCATTTAATGTGGATGAGAGGATGCTGGCCATAACCCCCCCCCCCTTCCATCCCTGCACTGGAAAATATTCTGGGCATCTGACAGTCTATAAGACCATGCTCTTCCCAGGTAGTTATCTTCTTATGACTGAGCACAGTTGGTTGATCTACTGCATACCTACACTAACTAGGTCAAAGTAGCATACACCCATCTGGAACCTATCAGTGACCATAGTCAACAGCAGGTGTACACACCCAGGCCAAGGGTGGAGCAGACCACTGTGGGATCTATGGAGATGCTTGAACGAGCAGATAACTAAGCCAAGCATGACTCAGAGTTTCTAGAGGCCACTAGGACTGCTGAGTAATACTTGGGGGTACAGAGTCAGAAAGGAAAGGACAAATGGTACCACTGATTAGGACCTCTGACGCTGTTTTCCCATCCTGTTTATTTGCCAAGTGACCCTGTGCCTGTTACCTTAAGTCAAAAACTTATGACTTTTCTGATGGGCAGTTTTTCCCCTGGTCTCCACTGGCCTGAAGAGACGTTTTCAAAGTGATGCTTTTCAGCTGAGGTGT ATTCCATCCTTCCCTCTGCTTCCACAGGTCTTGGATGGACAACCTCTGTTTGTTCCATTTATCTTTTTGCAGGTTTCTAGCCCCATCTAGGTGTCTGCTTCCAGGAGGACAAGAACTGGCATACATAACCATAAAGTCTCAACAACGCAGTAGGTGTTTTCCAGATATCCTCCCCTGAGTTCTTTCCAGCTGAATGAGGGAGCCACTGAGGTCTGTGCCTTTGCTTACCTCCCCAACTACCATGCAGCAGCCTTGACAACAGCACTTCTGTTCTGCTATGCAAATTTGTTTTCTCTCTCTACTGAATTCCCTGAAGGTAAGAACAGGAATTATCTTATCTCTAAACCAGGTATTGGGCAATTCAGGTGGATCATGCATGATACTCTGGGTCACACATCCCCC |
| RAD21  guide | CCTCAGATAATATG GAACCGTGG |
| RAD21^TST^  donor | CCCGGGTTGGAAGGTTATCAGGGGCCGGTTTGATTTTGGTTTTGTTTTCACTTTAAAATCTGCTGAGTGTTTGTTTTTGCTAACTCACATCTCTGTTGTGGCGCCTTCACTTACTTCTTGAACTCTGTATGCCTTAAGATAATATGCTTTAGATAATAAGGCCTTTACCTTAGTGACTAGCATGGGAACCACTTGCGTAACTACACGAATGTCCAGTCCCAGCTCCAGCCAAACGGAGCTCACTTTGACCAGTGCCAAAATTGCATCTTCTGGTTACTACTTTGTGCGTGAGTTACTTGAAATCATCTGCTTTGTTTTGTTCTATTTCAGCGAGCTCTTGCTAAAACTGGAGCAGAGTCTATCAGTTTGCTTGAGCTGTGTCGAAACACAAACCGAAAGCAGGCAGCAGCAAAGTTCTACAGCTTTTTGGTTCTTAAGAAGCAGCAAGCCATCGAGCTCACACAGGAAGAGCCGTACAGTGACATCATTGCAACGCCCGGCCCACGCTTTCACATCATCAGCGCCTGGAGCCACCCCCAGTTCGAGAAGGGCGGCGGCAGCGGCGGCGGCAGCGGCGGCAGCGCCTGGAGCCACCCCCAGTTCGAGAAGTGAGATATCGGAGCTAGATGTGTTCGAGCTAGTGATAACTCACTAGTACATACAAATTGCCCCCGTGTGCAGGGCACCAAAACCCTTTAAGAAAGTTTTTAGATTTCTGTTTGTACAAAAATCTTTGCCTTTTCTTTCTTCTTTTTCCCCCCAGTGTTTCTAATTTTGTCAACCATATTTTTAAGGGAAACTGCTTATTTGGGTTGGGTTTGTATTCCTGGAGAAAACAGTAGCCCAAGAACCCAGAAGACTTTTAACAGTTCAGAACAGATGTGTGCAATATTGGTGCATGTAAGAATATGGAGTAACAGTCAAAAGGCACCATTTTTAATGTTAGTTTTCCATTACTATGTTGAAAGGAAAACCTGCCTAGGAAAATGCCTGACACTTTAAGAACTGTGGTTTGAGTCCCTTGACAGGAAGAGAAAAATGTCTTCCCATCAGTGAAACCAACGGTCTGGTTAACCACTGTAGTAGGGATAGTGTGTGAAGCATCCCGGG |

**Table S3: Antibodies used for ChIP-seq experiments.**

| **Target** | **Supplier** | **Catalogue #** | **Lot #** |
| --- | --- | --- | --- |
| StrepTag | Qiagen | 34850 | - |
| CTCF | Merck Millipore | 07-729 | 2836926 |
| H3K4me1 | abcam | ab195391 | - |
| H3K4me1 | abcam | ab8895 | GR3206285-1 |
| H3K4me3 | Merck Millipore | 07-473 | - |
| H3K4me3 | abcam | ab8580 | GR3190162-1 |
| H3K27Ac | abcam | ab4729 | GB3205523-1 |
| RAD21 | abcam | ab1546769 | 1035529-6, 1035529-4 |
| RAD21 | abcam | ab992 | GR3310168-13 |

Table lists the antibodies used in the ChIP-seq experiments in this study, and lot numbers are provided if known. Specific information for each experiment is provided in the GEO submission (GSE244929).

**Table S4: Cell models**

| **Cell line** | **Species** | **Model name** | **Notes** |
| --- | --- | --- | --- |
| Erythroid  Day 10 CD34+ | Human | WT from 3 healthy donors (donor 1,2,3) | Used for ChIP-seq and ATAC-seq analysis.  Original protocol for CD34+ isolation: <https://doi.org/10.1038/s41467-022-31194-7> |
| Erythroid  Embryoid body derived | Mouse | WT  R2-only  SE-KO  R2-insertion | Used for ChIP-seq and ATAC-seq analysis. Capture-C and Tiled-C in WT and R2-insertion.  Original protocol for embryoid body differentiation: <https://doi.org/10.1371/journal.pone.0261950> |
| mESC E14 | Mouse | WT  R2-insertion | Used to perform Tiled-C for inactive enhancers, ChIP-seq, ATAC-seq, Capture-C and Tiled-C. |
| Erythroid  Ter119+ fetal liver derived | Mouse | WT | Used to perform Tiled-C for active enhancers.  Original protocol for harvesting ter119+ erythroid material: <https://doi.org/10.1038/nmeth.3664> |
| Erythroid  Ter119+ fetal liver derived | Mouse | R2-only | Used to perform Tiled-C for R2-only model enhancers.  Original protocol for harvesting ter119+ erythroid material: <https://doi.org/10.1038/nmeth.3664> |

Table detailing the origin and details of the various cell models used in this study.

**Table S5: Public data sources**

|  | **Cell type** | **GEO** | **Citation** |
| --- | --- | --- | --- |
| ATAC-seq | mESC | GSE94250 | Simon CS, Downes DJ, Gosden ME, Telenius J et al. Development 2017 Apr 1;144(7):1249-1260 |
| H3K27ac | mESC | GSE47950 | Wamstad JA, Alexander JM, Truty RM, Shrikumar A et al. Cell 2012 Sep 28;151(1):206-20 |
| H3K4me1 CTCF H3K27me3 | mESC | GSE30206 | Stadler MB, Murr R, Burger L, Ivanek R et al. DNA-binding factors shape the mouse methylome at distal regulatory regions. Nature 2011 Dec 14;480(7378):490-5 |
| H3K9me3 | mESC | GSE57092 | Bulut-Karslioglu A, De La Rosa-Velázquez IA, Ramirez F, Barenboim M et al. Mol Cell 2014 Jul 17;55(2):277-90 |
| H3K4me3 | mESC | GSE31039 | ENCODE |
| H3K4me1 | Ter119+ | GSE27921 | Kowalczyk MS, Hughes JR, Garrick D, Lynch MD et al. Mol Cell 2012 Feb 24;45(4):447-58 |
| H3K9me3 | Ter199+ | GSE36028 | ENCODE |
| ATAC-seq H3K4me3 H3K27ac CTCF H3K27me3 | Ter119+ | GSE97871 | Hanssen LLP, Kassouf MT, Oudelaar AM, Biggs D et al. Nat Cell Biol 2017 Aug;19(8):952-961 |

Table detailing the sources for the publicly available data used in this study.
